# Use of steroid and nonsteroidal anti-inflammatories in the treatment of rheumatoid arthritis: Systematic review protocol

**DOI:** 10.1097/MD.0000000000012658

**Published:** 2018-10-12

**Authors:** Mariana Del Grossi Moura, Luciane Cruz Lopes, Marcus Tolentino Silva, Sílvio Barberato-Filho, Rogério Heládio Lopes Motta, Cristiane de Cássia Bergamaschi

**Affiliations:** a Department of Pharmaceutical Sciences, University of Sorocaba, Sorocaba, São Paulo; b Faculty of Medicine, Federal University of Amazonas, Manaus, Amazonas; c Department of Pharmacology, Anesthesiology and Therapeutics, São Leopoldo Mandic Dental School and Research Center, Campinas, Brazil.

**Keywords:** corticosteroids, nonsteroidal anti-inflammatories, rheumatoid arthritis, steroid anti-inflammatories

## Abstract

**Background:**

Rheumatoid arthritis affects 1% of the world's population and its current treatment options are costly. There are not enough studies that evaluated the efficacy and safety of anti-inflammatory drugs medications used to reduce rheumatoid arthritis's symptoms. This study will evaluate the effectiveness and the safety of steroid and nonsteroidal anti-inflammatory drugs for the treatment of patients with rheumatoid arthritis.

**Methods:**

Randomized clinical trials eligible for our systematic review will enroll adults with rheumatoid arthritis treated with anti-inflammatory drugs compared with a control group (placebo or active control) at any dose, duration, and route of administration and double blind studies. In order to include all forms of rheumatoid arthritis and anti-inflammatory drugs, we will search the following electronic databases: Cochrane Central Register of Controlled Trials, MEDLINE (via Ovid); ExcerptaMedica Database (via Ovid); Cumulative Index to Nursing and Allied Health Literature (via Ovid); Web of Science; ClinicalTrial.gov; and WHO International Clinical Trials Registry Platform. We will not impose any language restrictions or publication status. Outcomes of interest include are pain, physical function, swelling, stiffness, grip force, radiological image of the joint, quality of life, adverse events, discontinuation due to adverse events, satisfaction with the treatment, and rescue medication for pain. A team of reviewers will independently screen search results, extract data from eligible trials, and assess risk of bias. We will use the Grading of Recommendations Assessment, Development and Evaluation approach to rate overall certainty of the evidence by outcome. Dichotomous data will be summarized as risk ratios; continuous data will be given as standard average differences with 95% confidence intervals.

**Results:**

The evidence derived by this study will increase awareness of the effectiveness and safety of steroid and nonsteroidal anti-inflammatory drugs for the treatment of rheumatoid arthritis.

**Conclusion:**

The results could guide patients and healthcare practitioners and help facilitate evidence-based shared care decision making.

## 1 Introduction

Rheumatoid arthritis (RA) is a chronic, autoimmune, and systemic inflammatory disease of unknown etiology, which mainly affects joints and is characterized by symmetrical synovial inflammation, resulting in destruction of joint cartilage, significant pain,^[1,2]^ and severe disability.^[3]^ RA affects 1% of the population^[4,5]^ and is more prevalent in women over 65 years.^[1]^

Arthritis in general has a significant impact on the quality of life of patients and society in terms of medical costs and disillusionment at work.^[6]^ The chronic inflammatory process in uncontrolled RA often results in functional disability. It is estimated that only 40% of these patients are able to work after 15 years of diagnosis. In addition to the associated morbidity, there is an increase in mortality; since the patients affected have a lower life expectancy compared the general population, mainly due to cardiovascular changes, the most common cause of death.^[7]^

Treatment of RA is based on pain relief, improvement of function, and prevention of joint damage.^[8]^ Despite the significant advances in disease management, a study conducted in Europe and the United States with 2795 adults with RA showed that although patients presented the disease at a controlled stage, most reported dissatisfaction with the level of pain, predominantly classified as moderate to severe.^[9]^

According to the American College of Rheumatology (ACR) and the European League Against Rheumatism (EULAR), the current approach focuses on disease early treatment with synthetic or biological disease-modifying anti-rheumatic drugs (DMARDs) as soon as the diagnosis is completed.^[10,11]^ The recommendation is to initiate the use of synthetic DMARD while the biological DMARD is usually recommended after its failure.^[12]^ It is recommended during the first 3 months after the diagnosis of RA.^[13]^

As adjunctive therapy in the treatment of RA, symptomatic drugs that act in the control of pain and inflammation such as analgesics, nonsteroidal anti-inflammatory drugs (NSAIDs), and steroids (corticosteroids) are recommended.^[14]^

NSAIDs inhibit cyclooxygenase enzymes (COX-1 and COX-2) and reduce pain and inflammation by restraining the formation of prostaglandins.^[15]^ Due to the reduction of prostaglandins production in the gastrointestinal mucosa, NSAIDs can cause gastric damage and compromise cardiovascular safety.^[16]^

Corticosteroids exert a potent anti-inflammatory effect. The recommendation is to use of a low-dose and short-term corticosteroid if the disease is classified as moderate or high activity, in conjunction with current therapy.^[11]^ The EULAR recommends the use of a low-dose corticosteroid as part of the initial treatment strategy in combination with DMARD for up to 6 months, decreasing the dose as clinically as possible.^[10]^

Considered as adjuvants in the treatment of RA, the literature has reported that the use of anti-inflammatories is of the common use in these patients^[17]^ and may bring benefit to the improvement of symptoms.^[10,18–20]^ Systematic reviews found benefit of using corticosteroids administered in addition to standard therapy in inhibiting the progression of radiological damage caused by RA^[21]^; however, they point to gaps regarding the effectiveness and safety of these drugs for the treatment of RA.

Systematic review published in 2004 found that the use of low-dose prednisolone (maximum 15 mg/d) was superior to placebo and NSAIDs in improving joint sensitivity and pain in patients with RA, but the authors reported some limitations of the study as poor description of adverse effects, substantial heterogeneity between clinical trials and restriction of findings only at the first month of treatment initiation.^[19]^

Another systematic review study also published in 2004 verified that NSAIDs were more effective and often more preferred than paracetamol by patients with RA; however, the low methodological quality of clinical trials included compromised the confidence in findings.^[22]^

Some clinical trials evaluated the efficacy of new corticosteroid formulations for the treatment of RA, as example of sustained release formulations^[23–25]^ and the intraarticular use of this class of drugs,^[26]^ in addition, the authors warned about the need for further studies evaluating aspects related to the safety of long-term use of these drugs.^[21]^

In view of this, this study aims to update the available evidence to verify the effectiveness and the safety of the use of steroid and NSAIDs for the treatment of RA by means of a systematic review and meta-analysis.

## 2 Methods

### 2.1 Standards

The systematic review will be performed according to the recommendations specified in the Cochrane Handbook for Interventional Reviews^[27,28]^ and reported according to the Preferred Reporting Items for Systematic Reviews and Meta-Analyses (PRISMA) statement.^[28,29]^

### 2.2 Protocol and registration

We registered our review protocol with the International Prospective Register of Systematic Reviews (https://www.crd.york.ac.uk/prospero/, PROSPERO- CRD42017073532). Ethical approval is not required because this is a literature-based study.

### 2.3 Eligibility criteria

#### 2.3.1 Inclusion criteria

Adults patients (>18 years old) with RA diagnosis according to the criteria of ACR^[30]^ or the equivalent criterion^[31]^ in treatment with steroid (*beclomethasone*, *betamethasone*, *budesonide*, *dexamethasone*, *flunisolide*, *fluticasone*, *fludrocortisone*, *hydrocortisone*, *methylprednisolone*, *prednisolone*, *prednisoneand triamcinolone*) and NSAIDs (*aceclofenac*, *acetylsalicylic acid*, *bufexamac*, *diclofenac*, *etodolac*, *fenclofenac*, *fenoprofen*, *flurbiprofen*, *ibuprofen*, *indomethacin*, *ketoprofen*, *ketorolac*, *meclofenamicacid*, *mefenamicacid*, *naproxen*, *niflumic acid*, *oxaprozin*, *oxyphenbutazone*, *phenylbutazone*, *piroxicam*, *sulfasalazine*, *sulindac*, *suprofen*, *tenoxicam*, *tiaprofenic acid*, *tolfenamic acid*, *nabumetone*, *celecoxib andetoricoxib*) at any dose, duration, and route of administration compared to placebo or active control. The type of study included will be randomized controlled trials (RCT) and double blind.

#### 2.3.2 Exclusion criteria

Studies in which more than 20% of patients have other disease, with sample below 200 and studies with participants with mild pain.

### 2.4 Measure outcomes

We will include studies that report any of the following outcomes.

#### 2.4.1 Primary outcomes

- decreased pain (visual analog scale [VAS] and other scales and patient global impression) in patients with initial pain moderate or severe;
- improvement of physical function (scales);
- decreased swelling (VAS and other scales);
- decreased stiffness (time in minutes or other scales);
- improvement of grip force (indicator of general strength and general health);
- progression of the disease through the radiological image of the joints; and
- improvement of quality of life (Short Form-36 and other scales).

#### 2.4.2 Secondary outcomes

- reports of adverse events including serious adverse events (that cause death, life-threatening, hospitalization, disability, or permanent damage);
- number of patients reporting any adverse effects;
- withdrawal of the study due to adverse events or treatment ineffectiveness;
- satisfaction with the treatment; and
- consume of rescue medication.

### 2.5 Search methods for primary studies

We will not impose any language restrictions or publication status.

#### 2.5.1 Electronic searches 

We will search the following electronic databases without publication status restrictions: Cochrane Central Register of Controlled Trials, MEDLINE; ExcerptaMedica Database; Cumulative Index to Nursing and Allied Health Literature; Web of Science; ClinicalTrial.gov; and WHO International Clinical Trials Registry Platform.

#### 2.5.2 Searching other resources

The grey literature will be identified by searching by reviewing the bibliographies of key papers and through contacts with appropriate experts and industry.

### 2.6 Search strategy

The search strategy will be comprised of both controlled vocabulary, such as the National Library of Medicine's Medical Subject Headings (MeSH) and keywords. The search strategy will be designed with the assistance of a trained librarian.

We will use the following MeSH terms, with associated keywords: intervention (anti-inflammatory agents); condition (arthritis Rheumatoid), and methodological filters will be applied to limit retrieval to RCT. The search strategy will be adapted for each database. MEDLINE (via Ovid) search strategy is provided in Table 1.

**Table 1:**
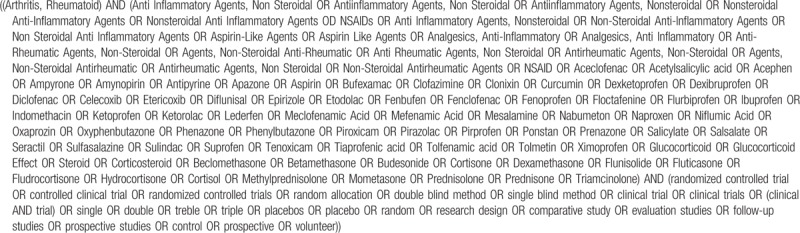
Search strategy for MEDLINE (via PubMed).

### 2.7 Eligibility determination

Six reviewers, working in pairs, will independently monitor potentially relevant citations and abstracts and apply the selection criteria. We will obtain full texts of any article that is considered eligible. The same reviewers will independently evaluate the eligibility of each full-text article. In case of duplicate publication, we will use the article with the most complete data.

The agreement between evaluators will be evaluated using the kappa coefficient (κ) of Cohen. Values of kappa between 0.40 and 0.59 will be considered to reflect fair agreement, values between 0.60 and 0.8 reflect good agreement, and values that are 0.75 or more reflect excellent agreement.^[32]^ Disagreement will be resolved through arbitration by a third-party investigator.

### 2.8 Data extraction

The same reviewers, working in pairs, will independently extract the data and will record information regarding patients, methods, interventions, outcomes, and missing outcome data using standardized and pretested data extraction forms with instructions. Before starting data abstraction, we will conduct calibration exercises to ensure consistency between reviewers. We will contact study authors to resolve any uncertainties. Disagreements will be resolved by consensus with any unresolved issues referred to another reviewer.

### 2.9 Risk of bias in individual studies

Using a modified version of the Cochrane collaboration risk of bias tool,^[28]^ the same pairs of reviewers will independently assess the risk of bias for each randomized trial, according to the following criteria: random sequence; allocation concealment; blinding of the patient, healthcare professionals, outcome assessors, data collectors, and data analysts; incomplete outcome data; selective outcome reporting; and major baseline imbalance. Reviewers will assign response options of “definitely yes,” “probably yes,” “probably no,” and “definitely no” for each of the domains, with “definitely yes” and “probably yes” ultimately being assigned a low risk of bias and “definitely no” and “probably no” a high risk of bias.^[33]^ Reviewers will resolve disagreements by discussion, and 1 arbitrator will adjudicate unresolved disagreements. For incomplete outcome data, loss to follow-up of <10% and a difference of <5% in missing data in intervention and control groups is considered low risk of bias.

### 2.10 Confidence in pooled estimates of effect

We will also independently rate the quality of evidence from randomized trials for each of the outcomes by using Grading of Recommendations Assessment, Development and Evaluation (GRADE) approach.^[33]^ In the GRADE approach, randomized trials begin as high-quality evidence but may be rated down by 1 or more of 5 categories of limitations: risk of bias, inconsistency, indirectness, imprecision, and reporting bias. The consensus will be established by discussion and by a third-party critic as needed. The final results will be summarized in an evidence profile.

### 2.11 Data synthesis

We will conduct analyses for each anti-inflammatory drug and for each outcome of interest. We will determine the confidence in estimates for each body of evidence and conduct an analysis for the body of evidence that warrants greater confidence.

Meta-analyses will be conducted using STATA software (version 14.2). We will use random-effects meta-analyses,^[34]^ which are conservative in that they consider within-studies and between-studies differences in calculating the error term used in the analysis.

For trials that report dichotomous outcomes, we will calculate the pooled relative risk with associated 95% confidence interval (CI). For continuous outcomes, we will use weighted mean differences (WMD) and its 95% CI as effect measure after we convert them into same scale. Once the WMD has been calculated, we will contextualize this value by noting, when available, the corresponding anchor-based minimally important difference (MID), the smallest change in instrument score that patients perceive is important.

If studies reported the same construct using different measurement instruments, we will calculate the standardized mean difference (SMD) as sensitivity analysis. The SMD expresses the intervention effect in standard deviation units, rather than the original units of measurement, with the value of an SMD depending on the size of the effect (the difference between means) and the standard deviation of the outcomes (the inherent variability among participants). For outcome measures that have an established anchor-based MID, we will use this measure to convert the SMD into an odds ratio and risk difference.^[35]^

To facilitate the interpretation of the effects of continuous outcomes, we will substitute the MID, when MID is available for different scales, for the standard deviation (denominator) in the SMD equation, which will result in more readily interpretable MID units instead of standard deviation units.^[36]^ If an estimate of the MID is not available, we will use a statistical approach developed by Suissa^[37]^ to provide a summary estimate of the proportion of patients who benefit from treatment across all studies. Statistical approaches to enhance the interpretability of results of continuous outcomes outlined in this paragraph will use methods cited as well as those described by Thorlund et al.^[38]^

The publication bias will be explored by statistical techniques (Egger and Peters tests). In both tests, we will consider as significant probabilities below 0.10.^[39]^ Another strategy will include visual inspection of the asymmetry in 2 funnel graphs (at least 10 studies contributed to a pooled analysis), obtained by sample size and logarithm of chance, and another by logarithm and standard error.^[40]^ Therefore, we will determine the smaller weight for studies with a small sample size, in order to avoid this type of risk of bias.

We will use recently developed approaches to address missing participant data for dichotomous outcomes^[39]^ and continuous outcomes.^[39]^ We will only apply these approaches to outcomes that show a significant treatment effect and report sufficient missing participant data to potentially introduce clinically important bias. Thresholds for important missing participant data will be determined on an outcome-by-outcome basis.

We will estimate heterogeneity associated with pooled effect estimates with the use of a chi-squared test and the I^2^ statistic.^[40]^ The following heterogeneity was considered: 0% to 25% (low heterogeneity); 50% (moderate heterogeneity); and 75% (high heterogeneity).^[41]^

We will also perform the meta-regression of the measures of outcomes identified in double-arcosene model of moments with the maximum likelihood restricted with the modification of the variance of the coefficients suggested by Knapp and Hartung.^[42,43]^ The coefficient (β), the probability (*P* value), and the residual heterogeneity will be calculated. Values of *P* < .05 will be considered significant.

Analysis of subgroups will be performed and possible explanations for heterogeneity will include the following: doses (higher vs lower) with an expected larger effect with higher doses, duration of the treatment (longer vs shorter) with an expected larger effect with longer duration of the treatment; risk of bias (high vs low) with an expected larger effect in trials at high or unclear risk of bias versus trials at low risk of bias, blinding (absence vs presence) with an expected larger effect in trials with absence blinding versus trials with blinding, and study size (large vs small studies) with larger studies provide better estimates of effect.

We will provide summary tables and a narrative synthesis if the meta-analysis is not appropriate due to excessive heterogeneity in populations, interventions, comparators, outcomes, or methodologies.

### 2.12 Summarizing evidence

We will follow the recommendation by the GRADE Working Group, presenting cumulative findings in evidence profiles.^[42,44]^ Evidence profiles provide succinct, easily digestible presentations of quality of evidence and magnitude of effects. The evidence profiles will be constructed with the following elements: a list of until 7 important outcomes, both desirable and undesirable; a measure of the typical burden of these outcomes (e.g., control group, estimated risk); a measure of the difference between risks with and without intervention; the relative magnitude of effect; numbers of participants and studies addressing these outcomes, as well as follow-up time; and a rating of the overall confidence in the estimate of effect for each outcome and comments, which will include the MID if available.

### 2.13 Ethics and dissemination

Ethical approval is not needed for a systematic review that does not involve privacy concerns due to collection or presentation of data from individual patients. The systematic review will be submitted to journals and presentations with scores in related research conferences.

## 3 Discussion

Our review will evaluate the available evidence for the treatment with steroid and NSAIDs for adult with RA, provide estimates of the effectiveness of treatments and their associated harms, and evaluate the quality of the evidence in a rigorous and consistent manner using the GRADE approach.^[43]^ The results of our systematic review will be of interest to public health and practitioners worldwide, particularly in Brazil.

The compiled information about these medications will inform patients and healthcare practitioners about their effectiveness and safety, and help facilitate evidence-based shared care decision making. This study will also identify key areas for future research.

### 3.1 Strengths and limitations of this study

- This systematic review will assess the effectiveness and the safety of the use of steroid and NSAIDs for the treatment of RA.
- The method of this review includes explicit eligibility criteria, comprehensive and extensive search in database, independent and paired evaluation to selection of studies.
- We will utilize robust statistical techniques and assess risk of bias of included studies. In addition, the GRADE approach will evaluate the strength and quality of the evidence body concerning the estimate of the effect for each outcome, including independent analysis of the risk bias, precision, consistency, publication bias, and indirect evidence.
- The quality of the primary studies to be included in this review may be a limiting factor if there is heterogeneity in study design, in doses, and in outcome measurements and thus they will have high bias risk. These limitations may decrease the quality of the evidence from the study findings regarding the effectiveness and safety of steroid and NSAIDs in RA.
- The results could guide patients and healthcare practitioners about the effectiveness and safety of the use of anti-inflammatory drugs and help facilitate evidence-based shared care decision making.

## Author contributions

Mariana Del Grossi Moura is the principal investigator and led the writing of the manuscript. Luciane Cruz Lopes and Cristiane de Cássia Bergamaschi are the project managers and coinvestigators and contributed to the writing and revision of the manuscript. Marcus Tolentino Silva, Sílvio Barberato-Filho, and Rogério Heládio Lopes Motta are coinvestigators and contributed to the writing and revision of the manuscript. All authors read and approved the final manuscript.

**Conceptualization:** Cristiane de Cássia Bergamaschi, Mariana Del Grossi Moura, Marcus Tolentino Silva.

**Methodology:** Cristiane de Cássia Bergamaschi, Mariana Del Grossi Moura, Luciane Cruz Lopes, Marcus Tolentino Silva, Sílvio Barberato-Filho, Rogério Heládio Lopes Motta.

**Project administration:** Cristiane de Cássia Bergamaschi.

**Writing – review & editing:** Cristiane de Cássia Bergamaschi, Mariana Del Grossi Moura, Luciane Cruz Lopes, Marcus Tolentino Silva, Sílvio Barberato-Filho, Rogério Heládio Lopes Motta.

**Writing – original draft:** Mariana Del Grossi Moura, Marcus Tolentino Silva, Sílvio Barberato-Filho, Rogério Heládio Lopes Motta.

Cristiane de Cássia Bergamaschi orcid: 0000-0002-6608-1806

## References

[1] Klarenbeek NB Kerstens PJ Huizinga TW Recent advances in the management of rheumatoid arthritis. BMJ (Clin Res ed) 2010;341:c6942. 10.1136/bmj.c6942 21177351

[2] Plum SM Park EJ Strawn SJ Disease modifying and antiangiogenic activity of 2-methoxyestradiol in a murine model of rheumatoid arthritis. BMC Musculoskelet Disord 2009;10:46. 19409094 10.1186/1471-2474-10-46 PMC2687416

[3] Wolfe F The epidemiology of drug-treatment failure in rheumatoid-arthritis. Baillieres Clin Rheumatol 1995;9:619–32. 8591645 10.1016/s0950-3579(05)80305-x

[4] Gabriel SE The epidemiology of rheumatoid arthritis. Rheum Dis Clin North Am 2001;27:269–81. 11396092 10.1016/s0889-857x(05)70201-5

[5] Alamanos Y Voulgari PV Drosos AA Incidence and prevalence of rheumatoid arthritis, based on the 1987 American College of Rheumatology criteria: a systematic review. Semin Arthritis Rheum 2006;36:182–8. 17045630 10.1016/j.semarthrit.2006.08.006

[6] Bergman MJ Social and economic impact of inflammatory arthritis. Postgrad Med 2006 5–11. 17960689

[7] Chung CP Oeser A Avalos I Utility of the Framingham risk score to predict the presence of coronary atherosclerosis in patients with rheumatoid arthritis. Arthritis Res Ther 2006;8:R186. 17169159 10.1186/ar2098 PMC1794532

[8] Smolen JS Aletaha D Koeller M New therapies for treatment of rheumatoid arthritis. Lancet 2007;370:1861–74. 17570481 10.1016/S0140-6736(07)60784-3

[9] Taylor P Manger B Alvaro-Gracia J Patient perceptions concerning pain management in the treatment of rheumatoid arthritis. J Int Med Res 2010;38:1213–24. 20925993 10.1177/147323001003800402

[10] Smolen JS Landewe R Breedveld FC EULAR recommendations for the management of rheumatoid arthritis with synthetic and biological disease-modifying antirheumatic drugs: 2013 update. Ann Rheum Dis 2014;73:492–509. 24161836 10.1136/annrheumdis-2013-204573 PMC3933074

[11] Singh JA Saag KG Bridges SL 2015 American College of Rheumatology guideline for the treatment of rheumatoid arthritis. Arthritis Rheumatol 2016;68:1–26. 10.1002/art.39480 26545940

[12] Katchamart W Trudeau J Phumethum V Methotrexate monotherapy versus methotrexate combination therapy with non-biologic disease modifying anti-rheumatic drugs for rheumatoid arthritis. Cochrane Database Syst Rev 2010;4:CD008495. 10.1002/14651858.CD008495 PMC8946299 20393970

[13] Ikeda K Cox S Emery P Aspects of early arthritis. Biological therapy in early arthritis-overtreatment or the way to go? Arthritis Res Ther 2007;9:211. 17540047 10.1186/ar2177 PMC2206357

[14] Emery P Treatment of rheumatoid arthritis. BMJ (Clin Res ed) 2006;332:152–5. 10.1136/bmj.332.7534.152 PMC1336767 16424492

[15] Whittle BJ COX-1 and COX-2 products in the gut: therapeutic impact of COX-2 inhibitors. Gut 2000;47:320–5. 10940262 10.1136/gut.47.3.320 PMC1728030

[16] Antman EM Bennett JS Daugherty A Use of nonsteroidal antiinflammatory drugs: an update for clinicians: a scientific statement from the American Heart Association. Circulation 2007;115:1634–42. 17325246 10.1161/CIRCULATIONAHA.106.181424

[17] Radner H Yoshida K Hmamouchi I Treatment patterns of multimorbid patients with rheumatoid arthritis: results from an international cross-sectional study. J Rheumatol 2015;42:1099–104. 26034147 10.3899/jrheum.141534

[18] American College of Rheumatology Subcommittee on Rheumatoid Arthritis Guidelines. Guidelines for the management of rheumatoid arthritis: 2002 update. Arthritis Rheum 2002;46:328–46. 11840435 10.1002/art.10148

[19] Gotzsche PC Johansen HK Short-term low-dose corticosteroids vs placebo and nonsteroidal antiinflammatory drugs in rheumatoid arthritis. Cochrane Database Syst Rev 2004;3:CD000189. 10.1002/14651858.CD000189.pub2 PMC7043293 15266426

[20] van Walsem A Pandhi S Nixon RM Relative benefit-risk comparing diclofenac to other traditional non-steroidal anti-inflammatory drugs and cyclooxygenase-2 inhibitors in patients with osteoarthritis or rheumatoid arthritis: a network meta-analysis. Arthritis Res Ther 2015;17:66. 25879879 10.1186/s13075-015-0554-0 PMC4411793

[21] Kirwan JR Bijlsma JW Boers M Effects of glucocorticoids on radiological progression in rheumatoid arthritis. Cochrane Database Syst Rev 2007;1:CD006356. 10.1002/14651858.CD006356 PMC6465045 17253590

[22] Wienecke T Gotzsche PC Paracetamol versus nonsteroidal anti-inflammatory drugs for rheumatoid arthritis. Cochrane Database Syst Rev 2004;1:CD003789. 10.1002/14651858.CD003789.pub2 PMC8730319 14974037

[23] Metselaar JM Wauben MH Wagenaar-Hilbers JP Complete remission of experimental arthritis by joint targeting of glucocorticoids with long-circulating liposomes. Arthritis Rheum 2003;48:2059–66. 12847701 10.1002/art.11140

[24] Metselaar JM van den Berg WB Holthuysen AE Liposomal targeting of glucocorticoids to synovial lining cells strongly increases therapeutic benefit in collagen type II arthritis. Ann Rheum Dis 2004;63:348–53. 15020326 10.1136/ard.2003.009944 PMC1754935

[25] Buttgereit F Doering G Schaeffler A Efficacy of modified-release versus standard prednisone to reduce duration of morning stiffness of the joints in rheumatoid arthritis (CAPRA-1): a double-blind, randomised controlled trial. Lancet 2008;371:205–14. 18207016 10.1016/S0140-6736(08)60132-4

[26] Garg N Perry L Deodhar A Intra-articular and soft tissue injections, a systematic review of relative efficacy of various corticosteroids. Clin Rheumatol 2014;33:1695–706. 24651914 10.1007/s10067-014-2572-8

[27] Better Health, Cochrane Collaboration. Cochrane: Trusted Evidence. Informed Decisions. 2015.

[28] Higgins JP Green S Cochrane Handbook for Systematic Reviews of Interventions. Vol. 4. Chichester, England: John Wiley & Sons Ltd; 2011.

[29] Moher D Shamseer L Clarke M Preferred reporting items for systematic review and meta-analysis protocols (PRISMA-P) 2015 statement. Syst Rev 2015;4:1. 25554246 10.1186/2046-4053-4-1 PMC4320440

[30] Arnett FC Edworthy SM Bloch DA The American Rheumatism Association 1987 revised criteria for the classification of rheumatoid arthritis. Arthritis Rheum 1988;31:315–24. 3358796 10.1002/art.1780310302

[31] Ropes MW Bennett GA Cobb S 1958 Revision of diagnostic criteria for rheumatoid arthritis. JBJS 1959;41:781–2. 13596783

[32] Guyatt GH Oxman AD Kunz R GRADE guidelines: 7. Rating the quality of evidence—inconsistency. J Clin Epidemiol 2011;64:1294–302. 21803546 10.1016/j.jclinepi.2011.03.017

[33] Akl EA Sun X Busse JW Specific instructions for estimating unclearly reported blinding status in randomized trials were reliable and valid. J Clin Epidemiol 2012;65:262–7. 22200346 10.1016/j.jclinepi.2011.04.015

[34] Montori V Ioannidis J Cook D Advanced topics in systematic reviews. McGraw-Hill, Fixed-Effects and Random-Effects Models. Users’ Guides to the Medical Literature: A Manual for Evidence-Based Clinical Practice. New York, United States of America: 2008.

[35] Busse JW Bartlett SJ Dougados M Optimal strategies for reporting pain in clinical trials and systematic reviews: recommendations from an OMERACT 12 workshop. J Rheumatol 2015;42:1962–70. 25979719 10.3899/jrheum.141440

[36] Johnston BC Thorlund K Schünemann HJ Improving the interpretation of quality of life evidence in meta-analyses: the application of minimal important difference units. Health Qual Life Outcomes 2010;8:116. 20937092 10.1186/1477-7525-8-116 PMC2959099

[37] Suissa S Binary methods for continuous outcomes: a parametric alternative. J Clin Epidemiol 1991;44:241–8. 1999683 10.1016/0895-4356(91)90035-8

[38] Thorlund K Walter SD Johnston BC Pooling health-related quality of life outcomes in meta-analysis—a tutorial and review of methods for enhancing interpretability. Res Synth Methods 2011;2:188–203. 26061786 10.1002/jrsm.46

[39] Akl EA Johnston BC Alonso-Coello P Addressing dichotomous data for participants excluded from trial analysis: a guide for systematic reviewers. PLoS ONE 2013;8:e57132. 23451162 10.1371/journal.pone.0057132 PMC3581575

[40] Higgins JP Thompson SG Quantifying heterogeneity in a meta-analysis. Stat Med 2002;21:1539–58. 12111919 10.1002/sim.1186

[41] Higgins JP Thompson SG Deeks JJ Measuring inconsistency in meta-analyses. BMJ (Clin Res ed) 2003;327:557–60. 10.1136/bmj.327.7414.557 PMC192859 12958120

[42] Guyatt GH Thorlund K Oxman AD GRADE guidelines: 13. Preparing summary of findings tables and evidence profiles—continuous outcomes. J Clin Epidemiol 2013;66:173–83. 23116689 10.1016/j.jclinepi.2012.08.001

[43] Guyatt GH Oxman AD Kunz R Rating quality of evidence and strength of recommendations: going from evidence to recommendations. BMJ 2008;336:1049. 18436948 10.1136/bmj.39489.470347.AD PMC2335261

[44] Guyatt GH Oxman AD Santesso N GRADE guidelines: 12. Preparing summary of findings tables—binary outcomes. J Clin Epidemiol 2013;66:158–72. 22609141 10.1016/j.jclinepi.2012.01.012

